# Supplementary material for: Evaluating socio-economic and conservation impacts of management: A case study of time-area closures on Georges Bank
Source: PLoS One. 2020 Oct 13;15(10):e0240322. doi: 10.1371/journal.pone.0240322 (PMC7553282; doi:10.1371/journal.pone.0240322)
Supplement: Attachment — Submitted filename: Cummings Review of Keith et al.docx [file pone.0240322.s001.docx]

Article Summary

The authors evaluate the impacts of scallop fishery time-area closures to the scallop fishery and to the species of conservation concern, Atlantic cod and yellowtail flounder. Because a monitoring program directed at evaluating these impacts does not exist the authors use scallop fishing effort, location, and discard (of cod and yellowtail) data to evaluate the impacts. They conclude that closures are not impacting the socioeconomics of the scallop fishery, and that conservation efforts are not being achieved.

Overall Review

I conclude that the data used by the authors is sufficient and supports the authors conclusions with regards to the impact of closures on the scallop fishery, but that the data is insufficient to evaluate the conservation impacts of the closures on cod and yellowtail stocks in the manner the authors intend. Therefore, I suggest a major revision with regards to the conservation objective.

The discard data, as with any harvest data, is an index or proxy for the population status. Assessing the status of a population from an index without accounting for effort, detection, selectivity, and other data sources provides a limited picture of population status. Additionally, the conservation impact measured using the discard data is not the change in discard from pre-closure to closure eras, but time periods with-in the closure era. This doesn’t give a picture either of the current conservation status of the populations, or the effect of closures on discarding.

I suggest two paths forward for the authors. A) Be clearer that the objective regarding conservation impacts is not to assess the conservation status of cod and yellowtail, but only to assess the impact of scallop fisheries on these populations irrespective to the underlying health of those populations. That is, only draw conclusions on the amount of discarding that is occurring and note methods to further reduce discarding rather than draw conclusions on what the discarding means to the conservation status of species of concern B) Include and evaluate additional data sources regarding the underlying conservation status of cod and yellowtail and evaluate the impacts of discards in this light.

For example, the authors conclude that the closure period is insufficiently long and insufficiently large in area because the proportional discard rate in the time periods before and after the closure are higher than the median rate for the remainder of the year and discarding during the closure period is not reduced relative to the discard rate immediately outside of this closure period. However, these things could be true despite the closure having an impact, and an impact sufficient to sustain the populations of concern. Whether populations are conserved can only be assessed through assessment of the actual status of the populations. Put another way, why would someone care if some discarding is still occurring if the conservation objective is being achieved despite discarding?

Path A for this example would entail retracting the conclusion that the pattern of discards provides information on achievement of conservation objectives. While it does provide some information on the relative amount of discarding, discarding, and discarding by time-period, is an insufficient proxy to measure conservation objectives. Especially the broad unspecified conservation objective discussed here without any associated performance measures. Path B) utilize addition data about the productivity and abundance of cod and yellowtail populations through time to determine how influential the impact of the discards is on the status of the population, and the populations likelihood of achieving conservation performance metrics.

There are also a number of minor changes to figures that would ease interpretation of the results.

Specific Comments

Line 32-34

Awkward phrasing. Maintaining populations above biological reference levels does not maximize socio-economic benefits. Also, biological reference levels, aka biological reference points are a management tool rather than a statement of an objective.

Lines 56-59

Monitoring is not required for effective management, nor are indicators. Without uncertainty in a system, given objectives a management plan could be developed and implemented without the need for monitoring or indicators because the management outcome would be known. Indicators and monitoring are only necessary if there is enough uncertainty in the system that learning can occur from monitoring and that learning will provide a greater benefit than the cost of conducting the monitoring.

Line 66

Components rather than component

Lines 88-90

Presuming the reason for the conservation concern is solely the fishery this would be the case. However, fishing impact is not the only cause for concern for many species of conservation concern, especially those that are primarily by-catch fisheries.

Line 90-91

Suggest rephrasing as “Successful SFM strategies will combine minimal socio-economic impact with maximal conservation impact” because for example, strategies that are worth implementing could be “unacceptable” socio-economically and yet have enough conservation impact to offset them, and vice versa.

Lines 196-199 & 212-217

Why was this data binned into categories, and why were these categories chosen? Unless there was a need for binning, I suggest working with the natural scale data.

Lines 243-245

What about the data was insufficient?

Lines 271-288,306-311, Figure 4

What is the importance of this north-south division? As a reader unfamiliar with SFAs, are northern and southern areas different SFAs. Please clarify, and either way, why would someone care differently about northern and southern areas?

Lines 277-281, Figure 2

Suggest producing an additional pair of images (e and f) showing the proportional effort in an area relative to the proportional productivity (i.e. color scale = proportional effort – proportional productivity)

Lines 291-293

This is the opposite result from the hypothesis that closures are detrimental to scallop fishing. Address this in the discussion. Has the fishery gotten better at finding scallops, did closures have an impact on the fishing behavior that could explain this?

Lines 294-301, Figure 3

If the intended take-away from this figure is that effort is allocated more proportionally to total biomass I suggest replacing the figure with a metric such as Effort deviation = sum across cells(proportional effort – proportional productivity) by era. Could also be calculated for cod and yellowtail areas by era. This would contain more complete information that the % by quadrant.

Line 303-304, 313-314

Reporting % of total productivity in closed cells rather than proportion that are high productivity would provide more information.

Lines 306-311, Figure 4

Either note that the y-axis lower bound is not zero, or alter the bounds on the y-axis to extend to zero.

Lines 323-325, Figure 5

Lower than what? Complete the sentence “…was lower…“ with “*than…*” For example, “*during the pre-closure era*”, or “*during the closure*”. It isn’t clear from the figure, so why is Figure 5 referenced on this line? If during the closure, it doesn’t appear so in Figure 5 where the before and during effort look roughly equal.

Line 338

To me there is no need to reference Figure 5 here, those points are not perceptibly above 0

Line 348

How much has effort throughout the region declined? How has the effort on Georges Bank changed, not as a percentage of regional effort, but absolutely?

Figure 7

If it doesn’t make the figure too difficult to read include the proportion by month as well as the cumulative proportion

Lines 363-364

This implies a major reduction in the impact of fishing on these populations, and therefore potential achievement of the conservation objectives.
That is, unless there was also a 10-fold decline in the cod and yellowtail populations. Another additional way to state the degree of discard would be to report the proportion of the populations lost due to discarding in the scallop fishery. This would provide the missing information on the impact of discards to the populations.

Lines 366-368

It appears it is above average for the three months after the closure for cod and three months before and after for yellowtail.

Figure 8

1. Why do most of the observed data points, way more than 5%, fall outside of the 95% confidence interval?
2. The data in c and e is the same, just transformed. Same for d and f. However, the curves fit in image c and e appear very different. Rather than attempting to fit a spline to these point that assumes some degree of correlation to neighboring months just show the medians for each month.

Lines 398-399

The results show fishing shifted to more productive areas during the closure era, a positive correlation between time-area closures and socio-economic objectives as expressed through the metric of effort allocated to more productive locations.
The results show a 10-fold decrease in discard weight, and a seasonal pattern of discarding weight and rate. Neither provides information on the abundance or productivity of the populations of conservation concern. Therefore, because both the conservation objectives are not specified, and a reduction of discard weight from the scallop fishery presumably is the goal of closures in terms of achieving conservation, the results do not support the conclusion that time-area closures did not achieve conservation objectives.

Line 400-401

The methods here leave out a key step of specify objectives. Without clear objectives performance metrics that measure the status of those objectives will be lacking and monitoring may not measure the parameters of value.

Lines 420-423

Excellent point!

Line 422

Add “in”, to obtain “…coincidental changes *in* the state…”

Line 427-429

An important note!

Line 433-435

This presumes the amount of discarding would have to be anomalous to be effective. This may not be the case because, 1) effective is not defined, and 2) the discarding that would have resulted without the closure is unknown. Perhaps there would have been an anomalously large amount of discarding during those time periods without the closures. Finally, in the case of yellowtail where fishing seemingly would occur outside of closures anyway, an objective of educating the fishing community about where and when yellowtail spawn to create a conservation mindset, or to practice should the population recover and therefore be subject to additional fishing pressure, could also be an objective and affect the conservation of the population in other ways.

Line 440-443

Enough and adequate are value statements that have not been defined and no information is provided about the spatial distribution of cod or yellowtail. This conclusion is not supported. The only conclusion that can be drawn from the data provided is that the closures do not eliminate by-catch and discarding of cod and yellowtail. However, that amount of reduction in by-catch may be more than enough or exceed what is adequate.

Line 465-466

Declines *in* yellowtail biomass

Lines 457

“Despite incomplete elimination of discarding during these closures, …”

Line 458-459

Yes.

Line 459-466

How low is the biomass of these populations, and have they declined by an order of magnitude? Discards below the quota seems like an indicator of closure success rather than an indicator of a population’s conservation status.

Line 492-494

Remove inaccurate concluding sentence, or restate that extending the duration of the closures may reduce by-catch by capturing a greater portion of the period when cod and yellowtail populations occupy Georges Bank.

Line 495-496

“To be *maximally* effective…”

Line 496-497

Remove. Without a pre-post comparison the impact on the discard risk is unknown. Alternatively, have not … eliminated the discard risk.

Line 497-499

Therefore, *because these populations have not achieved conservation targets …*

Line 521-525

This information is crucial to interpreting the discard data presented in the results and should not be withheld until the final sentence of the discussion. For example, if the effort from pre to post closure reduced by 50%, then the 10-fold reduction in discard weight is a 5-fold reduction in discard rate per unit of effort.

Line 527-529

Remove “conservation impact”, or rephrase as “*the degree of discarding unaffected by the current closure practices*”

Line 528-529
“establishment *of* a dedicated *~~to~~* monitoring program”

Line 529-531

Remove “the spatial and temporal extent of these closures were insufficient to protect either species during spawning and” or rephrases as “*insufficient to eliminate by-catch*”

Line 531

replace “significant” with “*consequential*” or the like

Line 532-533

What is the evidence for this?
