# Supplementary material for: Evaluating socio-economic and conservation impacts of management: A case study of time-area closures on Georges Bank
Source: PLoS One. 2020 Oct 13;15(10):e0240322. doi: 10.1371/journal.pone.0240322 (PMC7553282; doi:10.1371/journal.pone.0240322)
Supplement: Attachment — Submitted filename: Response to reviewers_final.docx [file pone.0240322.s002.docx]

**RESPONSES TO EDITOR AND REFEREES**

**A) Editor – PLOS One Comments to the Author:**

1- After careful consideration, we feel that it has merit but does not fully meet PLOS ONE’s publication criteria as it currently stands. Therefore, we invite you to submit a revised version of the manuscript that addresses the points raised during the review process. In particular, please respond to Reviewer #2’s significant concerns about the validity of conclusions drawn about achieving conservation objectives. Also, in the response to PLOS ONE’s Data Sharing question, please be specific about which data can and cannot be shared publicly. The current response is: “Some of the data cannot be shared publicly because of privacy restrictions and collaborative agreements with entities which collected the data.”

- **RESPONSE:** **Thank you. The manuscript has been revised throughout in accordance with the reviewers’ recommendations. In particular, we focused on clarifying the conservation objectives of the closures as was suggested in Path A) from the second reviewer. In our cover letter we have clarified the legal limitations on data sharing in our submission, these are largely related to restrictions due to the Canadian *Privacy Act*; however, we have clarified where the data resides and how to request these data, although in the case of commercial data there may be restrictions due to privacy considerations.**

**B) REVIEWER 1: COMMENTS TO AUTHORS**

2- In their manuscript, the authors describe a very interesting application of fishery dependent and independent data to assess the socio-economic and conservation impacts of time-area closures on Georges Bank. This paper was a pleasure to read and I appreciated the general implication that closures need to be carefully planned and monitored to ensure they are effective. Unfortunately, as the authors point out, closures are sometimes initiated without a plain for monitoring their success. Despite this common reality, the manuscript provides some hope that practitioners can assess the impacts of closures using data from common fisheries monitoring programs. In their case study, several data sources were utilized to assess the impacts of the closures on the fishery (logbooks, catch statistics, VMS, scallop surveys) and the results from several careful analyses show that there are no clear impacts. The conservation implications of the work was also clear, however, the analyses were hampered to a degree by the duration, spatial resolution and coverage of the observer program. For instance, if the series were longer, the authors could have compared bycatch rates in the pre and post closure eras. This limitation precluded the use of a control within the same space. I wonder, however, if the authors considered comparing bycatch rates on the Georges Bank with those from other banks? While this would not control for spatial confounds, it could be an informative comparison. I am also curious whether maturities are ever recorded for bycaught cod or yellowtail as the objective is to minimize bycatch of spawning individuals. Finally, are bycaught species recorded in the logbooks? Apologies for all the questions. I realize that my curiosity may not be helpful here and I suspect the authors have explored these ideas and know that the data are either not available or they are unreliable. I simply raise these questions, just in case, as they may help strengthen their argument that the conservation objectives are not being met.

   Data limitations are, of course, no fault of the authors and they only bolster the importance of planning and coordinating monitoring efforts to ensure the objectives of a closure can be evaluated. I reiterate that the authors have done a great job pulling together multiple data streams into one cohesive applied analysis. My only general comment is that the authors should add a little more detail to their model descriptions; in particular, please clarify the unit of analysis. For equations 5 and 6, I could not discern the level of aggregation of the response - does i = vessel, month? For equation 12, I think i = year. Below I provide several specific comments:
   - **RESPONSE: We thank the referee for their constructive criticism and thoughtful perspective. Unfortunately, there is very limited observer coverage on the other banks (1 trip per year) and a comparison with these banks would have little utility. We are not aware of any maturity data being collected from the observer program for either species, but we agree this would be very valuable information to collect during trips in these months. The logbooks from the scallop fishery do not record bycatch of either of these species. We have clarified the text around equations 5 and 6.**
3- Line 24 - Consider removing "and there was no evidence of recovery in either stock" as it implies that there is a link between discards and cod and yellowtail trends (this is ruled out in the discussion).

- **RESPONSE: Excellent point, removed**

4- Line 64 - Consider replacing "SFM objectives" with "both socio-economic and conservation objectives".
   - **RESPONSE: Revised**
5- Line 96 - I presume bycatch should be added here: "...are small annual [bycatch] quotas allocated..."
   - **RESPONSE: It’s slightly more nuanced than this, as it is a bycatch reserve. Cod and Yellowtail are transboundary assessed species between Canada and the US where the stocks are shared between the countries. From Canada’s share of the cod and yellowtail TACs, Canada sets aside a bycatch reserves from its TACs to cover bycatch on the Canadian side of Georges Bank. Mortality of discards for cod and yellowtail in the offshore scallop fishery is expected to be 100 percent and therefore it is treated as “catch” against this bycatch reserve. We have revised the wording for this to more accurately reflect this nuance.**
6- Lines 131-143 - Consider combining these two paragraph. I initially assumed that the first paragraph was focused on cod and the second on yellowtail but was confused by Lines 139-143..
   - **RESPONSE: Revised to put cod/yellowtail in one paragraph and then have a second paragraph starting with ‘DFO Resource…’**
7- Line 141 - Spell out the area in km^2^ or add a column to Table 1 for the number of cells.
   - **RESPONSE: Revised the table caption to include the area of each cell.**
8- Line 145 - Consider adding SFA to Figure 1 as some boundaries are mentioned in other figures and throughout the methods..
   - **RESPONSE: Revised throughout for clarity**
9- Lines 163-164 - What is the percent coverage of the observer program, or how many scallop fishing trips are conducted per month?
   - **RESPONSE: The target coverage is 2 trips per month on Georges Bank (see line 164 in original manuscript), this currently corresponds to approximately 12% per year based on effort. Text updated .**
10- Line 191 - "...total time spent [fishing] within...".
    - **RESPONSE: Revised**
11- Line 207 onward - This is perhaps semantics, but when I read productivity I think of growth, recruitment and mortality. Looking at what is presented, I tend to think biomass or density. Perhaps this is a fishy way to think and, for scallops, high productivity is implied by high density.
    - **RESPONSE: This is a good point, the difference here is really due to scallop being essentially sessile and as and such if an area contains high biomasses (especially in light of fishing effort) then this area has a combination of high growth, high recruitment, and low mortality which leads one to be able to identify high productivity regions. Revised to clarify that scallop are a bit unique here**
12- Lines 264-265 - While it is clear from Figure 8 that there is no clear precipitous decline in bycatch rates during the closures, I wonder if the GAM may be smoothing over a very minor impact. The closed areas are not fished so some, albeit minor, bycatch may be avoided. As the authors note, the crux of the issue is that the size of the closed area is small and the duration too short. Because of this, another covariate is not going to change the story. Nevertheless, consider adding timing as an explanatory variable to get an estimate of the difference in the means. Also consider adding area closed as an explanatory variable as bycatch rates should be lower when more area is closed (then again, there is probably insufficient interannual variability in the area protected to detect a minor signal). Finally, it may be better to use a cyclic cubic regression spline for the smooth for month (i.e. `s(month, bs = "cc")`).
    - **RESPONSE: These are all excellent considerations which we will address in turn.**
      i. **The timing of the closures is very similar between years, the yellowtail dates are the same every year, while the cod closure opening dates have generally varied by several days. This analysis would not be doable within the context of Figure 8, moreover we would envision this as some sort of regression analysis looking at the effect of duration on some discard metric from those months, even with the outlier 2007 season the relationship is non-significant (see Fig R1 below, p = 0.06).**
      ii. **You are correct that the inter-annual variability in the area closed is too small see a signal, this is especially problematic for Yellowtail in which the area of the closures has not changes for the last several years. We show this analysis for cod in Fig R2 (p = 0.11)**
      iii. **With respect to using a cyclic cubic regression, the reason we decided not to use this is that the start of the fishing season is a reset in which the fishery behaves quite differently then at the end of the year in which they have already captured most of the quota, different components of the fleet are fishing in different areas. You can see these difference best in the discard rate anomaly figure in which the discard rates in January are generally much higher than in December, by using a cubic spline we end up pulling up the end of the year and pulling down the beginning of the year in a way that doesn’t align with the underlying data.**
    - **Fig R1: Discard rate anomaly vs closure duration (number of days) for Cod in February and March, relationship is marginally significant (P = 0.06) and this relationship is strongly influenced by the 2007 data which has high leverage. Note that it would be month 2 (February) that is impacted by a delayed opening of the closure. Month 3 has always had the closure in place for the duration of the month. Symbols are Month-Year, e.g. March 2017 is 3-17.**

- **Fig R2: Discard rate anomaly vs Area for Cod in February and March, relationship is not significant (P = 0.11).**

13- Line 328 - Might be informative to report some statistics here, such as the parameter estimates of the difference between two groups plus/minus 95% confidence intervals.
    - **RESPONSE: We did have this reported at one point during the drafting of the manuscript but it was removed as we felt that it took away from the text as it made the text difficult to read/follow. If it is strongly felt that this should be included we think this would work better as a table, but this again is repetitive with Figure 5.**
14- Lines 332-336 - Drop the text stating that effort declined slightly because it looks like there is no clear difference in effort on the bank before, during and after the yellowtail closure.
    - **RESPONSE: Revised**
15- Lines 337-338 - The points in Figure 5 for the during-closure period look very close to zero with high confidence. Perhaps the reference to Figure 5 is not needed here because it is not easy to confirm the assertion given the figure; the percentages from the VMS data serve the purpose.
    - **RESPONSE: Revised**
16- Lines 408 and 413 - It may be more relatable to simply state the percentages of the landings taken during the cod and yellowtail closures rather than specific quarters or one month.
    - **RESPONSE : Good point. Note that for the Yellowtail closures June is equivalent to the closure period which is noted in the text. Text and numbers revised throughout this section.**
17- Lines 516-525 - When was this informal program initiated?
    - **RESPONSE: While there is no formal documentation about when this program began, we believe it was around 2008 based on information in this fisheries initial MSC documentation.**
18- Lines 528-529 - Suggested tweak of the wording here: "...and conservation impacts of time-area closures even without the establishment of a dedicated monitoring program.".
    - **RESPONSE: Thanks for catching that typo.**
19- Figure 1 - Add scallop fishing areas if it does not make the map too busy.
    - **RESPONSE: Revised in the text and figure legend for clarity**
20- Figure 4 - Consider shifting this figure to an appendix.
    - **RESPONSE: We have considered this and felt that to be consistent with the rest of our analyses this figure should remain in the main text but we are certainly open to this suggestion if you feel strongly about moving this figure.**
21- Figures 6 and 7 - Shaded areas indicating closure windows may be useful in these plots as well.
    - **RESPONSE: Good idea, done!**

**C) REVIEWER 2: COMMENTS TO AUTHORS**

22- I conclude that the data used by the authors is sufficient and supports the authors conclusions with regards to the impact of closures on the scallop fishery, but that the data is insufficient to evaluate the conservation impacts of the closures on cod and yellowtail stocks in the manner the authors intend. Therefore, I suggest a major revision with regards to the conservation objective.

    The discard data, as with any harvest data, is an index or proxy for the population status. Assessing the status of a population from an index without accounting for effort, detection, selectivity, and other data sources provides a limited picture of population status. Additionally, the conservation impact measured using the discard data is not the change in discard from pre-closure to closure eras, but time periods with-in the closure era. This doesn’t give a picture either of the current conservation status of the populations, or the effect of closures on discarding.

    I suggest two paths forward for the authors. A) Be clearer that the objective regarding conservation impacts is not to assess the conservation status of cod and yellowtail, but only to assess the impact of scallop fisheries on these populations irrespective to the underlying health of those populations. That is, only draw conclusions on the amount of discarding that is occurring and note methods to further reduce discarding rather than draw conclusions on what the discarding means to the conservation status of species of concern B) Include and evaluate additional data sources regarding the underlying conservation status of cod and yellowtail and evaluate the impacts of discards in this light.

    For example, the authors conclude that the closure period is insufficiently long and insufficiently large in area because the proportional discard rate in the time periods before and after the closure are higher than the median rate for the remainder of the year and discarding during the closure period is not reduced relative to the discard rate immediately outside of this closure period. However, these things could be true despite the closure having an impact, and an impact sufficient to sustain the populations of concern. Whether populations are conserved can only be assessed through assessment of the actual status of the populations. Put another way, why would someone care if some discarding is still occurring if the conservation objective is being achieved despite discarding?

    Path A for this example would entail retracting the conclusion that the pattern of discards provides information on achievement of conservation objectives. While it does provide some information on the relative amount of discarding, discarding, and discarding by time-period, is an insufficient proxy to measure conservation objectives. Especially the broad unspecified conservation objective discussed here without any associated performance measures. Path B) utilize addition data about the productivity and abundance of cod and yellowtail populations through time to determine how influential the impact of the discards is on the status of the population, and the populations likelihood of achieving conservation performance metrics.

    There are also a number of minor changes to figures that would ease interpretation of the results.
    - **RESPONSE: We also thank this referee for their constructive criticism and thoughtful perspective. We apologize for the confusion with what was meant by the ‘conservation objectives’. We wholehearted agree that we cannot infer anything about the impact of the closures on the population status of either cod or yellowtail flounder from this kind of analysis; that would be a significant overreach of our data and analysis. The “conservation objectives” that we were assessing in this analysis was whether the closures reduced discards of yellowtail and cod during spawning (see lines 97-99 in original manuscript). We were not attempting to address whether the larger conservation objectives of rebuilding either stock was impacted by these closures. We have followed your Path A) to clarify what the conservation objectives of the closures were. This has resulted in numerous revisions throughout the manuscript to clarify our objectives.**
23- Line 32-34 Awkward phrasing. Maintaining populations above biological reference levels does not maximize socio-economic benefits. Also, biological reference levels, aka biological reference points are a management tool rather than a statement of an objective.
    - **RESPONSE: Revised.**
24- Lines 56-59 Monitoring is not required for effective management, nor are indicators. Without uncertainty in a system, given objectives a management plan could be developed and implemented without the need for monitoring or indicators because the management outcome would be known. Indicators and monitoring are only necessary if there is enough uncertainty in the system that learning can occur from monitoring and that learning will provide a greater benefit than the cost of conducting the monitoring.
    - **RESPONSE: We feel (see references in this section) that there is a great deal of literature suggesting that monitoring is crucial for effective management. We are unaware of any fisheries systems in which there is not a significant amount of uncertainty.**
25- Line 66: Components rather than component
    - **RESPONSE: Revised, thanks for catching that.**
26- Lines 88-90: Presuming the reason for the conservation concern is solely the fishery this would be the case. However, fishing impact is not the only cause for concern for many species of conservation concern, especially those that are primarily by-catch fisheries.
    - **RESPONSE: We are referring to any fishery impact here, not just a directed fishery, for example in this study the conservation objectives are tied to bycatch.**
27- Line 90-91 Suggest rephrasing as “Successful SFM strategies will combine minimal socio-economic impact with maximal conservation impact” because for example, strategies that are worth implementing could be “unacceptable” socio-economically and yet have enough conservation impact to offset them, and vice versa.
    - **RESPONSE: We believe that a successful strategy could have a large socio-economic impact, but the conservation impact would be enough that it is acceptable socio-economically. If a strategy is unacceptable socio-economically we would argue that it will not be implemented by management, thus why we used the language ‘acceptable’ in this context.**
28- Lines 196-199 & 212-217. Why was this data binned into categories, and why were these categories chosen? Unless there was a need for binning, I suggest working with the natural scale data.
    - **RESPONSE: The analysis was originally done on the natural scale but using that scale makes the discussion of the results very complex to interpret, especially given that it is actually the values relative to each other that matter in this story. The decision to bin came after a significant amount of discussion between the authors and some advice from other collaborators less familiar with these data. For example, the reader is not particularly concerned if the biomass of scallop is 4 or 5 tonnes/km^2^, simply whether this is a high value relative to other areas. As mentioned in the methods, for the effort data the quantiles were simply binned into low, below median, above median, and high effort bins. For the scallop biomass the average cell biomass was chosen as the central point indicating areas on the bank of average productivity, while high biomass cells where chosen as the cells with the top 25% of scallop biomass. Three bins were chosen as we know that the scallop fishery does not regularly fish cells with below average biomass and that these cells make up a small percentage of the biomass on the bank while covering a large area on the bank. These decisions were based on our understanding of scallop and the fishery and our ability to tell the story in a relatively straight forward manner for the reader. We have revised the text to be clearer as to the method used to explain the binning.**
29- Lines 243-245 What about the data was insufficient?
    - **RESPONSE: The first full year of observer data collection was 2005. In 2005 there was no observer coverage in February or June, so no information is available for the Yellowtail closure in 2005, and the only information which informs rates during the closure windows is from March in 2005 (this datum indicates a relatively low discard rate). In 2006 there is information from June (recall the yellowtail closures did not commence until 2007; this datum indicates a high DR in this month). So there is 1 datum available for the cod closure and 1 datum available for yellowtail closure from the pre-closure which we consider insufficient to include in this analysis. Additional text has been added to clarify this.**
30- Lines 271-288,306-311, Figure 4 What is the importance of this north-south division? As a reader unfamiliar with SFAs, are northern and southern areas different SFAs. Please clarify, and either way, why would someone care differently about northern and southern areas?
    - **RESPONSE: Added to Figure 1 and a sentence in the methods explaining that the northern half of the bank has been a higher productivity region for scallop over the last two decades.**
31- Lines 277-281, Figure 2 Suggest producing an additional pair of images (e and f) showing the proportional effort in an area relative to the proportional productivity (i.e. color scale = proportional effort – proportional productivity)
    - **RESPONSE: While we can certainly do this, it is unclear what additional information this would bring to the analysis as similar information is contained in the above figures. Also, we have revised Figure 3 as suggested in comment 33 which contains a summary of this information that is likely more relevant to the reader.**
32- Lines 291-293 This is the opposite result from the hypothesis that closures are detrimental to scallop fishing. Address this in the discussion. Has the fishery gotten better at finding scallops, did closures have an impact on the fishing behavior that could explain this?
    - **RESPONSE: Agreed that this is consistent with the closures having no significant impact on the fishery. We have added a sentence to the discussion to highlight this. The reasons for the increased efficiency are outside the scope of this paper, but have to do with more efficient vessels and high quality bottom mapping.**
33- Lines 294-301, Figure 3 If the intended take-away from this figure is that effort is allocated more proportionally to total biomass I suggest replacing the figure with a metric such as Effort deviation = sum across cells(proportional effort – proportional productivity) by era. Could also be calculated for cod and yellowtail areas by era. This would contain more complete information that the % by quadrant..
    - **RESPONSE: Great idea, we decided to do a slight variant of this and report the percentage of effort and percentage of biomass in each quadrant of the figure to provide a more comprehensive summary of the quadrants.**
34- Line 303-304, 313-314 Reporting % of total productivity in closed cells rather than proportion that are high productivity would provide more information.
    - **RESPONSE: Given the relatively small size of the closures we feel identification of the cells that are high productivity is the more easily interpreted metric, our point here is that cod cells relatively frequently overlap with high scallop productivity whereas yellowtail cells do not frequently overlaps with high scallop productivity. We feel the proportion of the total bank biomass inside these cells is a less intuitive metric.**
35- Lines 306-311, Figure 4 Either note that the y-axis lower bound is not zero, or alter the bounds on the y-axis to extend to zero.
    - **RESPONSE: Revised**
36- Lines 323-325, Figure 5 Lower than what? Complete the sentence “…was lower…“ with “than…” For example, “during the pre-closure era”, or “during the closure”. It isn’t clear from the figure, so why is Figure 5 referenced on this line? If during the closure, it doesn’t appear so in Figure 5 where the before and during effort look roughly equal.
    - **RESPONSE: Agreed, this paragraph was revised for clarity.**
37- Line 338 To me there is no need to reference Figure 5 here, those points are not perceptibly above 0
    - **RESPONSE: Revised**
38- Line 348 - How much has effort throughout the region declined? How has the effort on Georges Bank changed, not as a percentage of regional effort, but absolutely?
    - **RESPONSE: See lines 520-522 in original text, in recent years is it half what was observed in the early 2000’s yet the quota has been relatively stable during this period.**
39- Figure 7 If it doesn’t make the figure too difficult to read include the proportion by month as well as the cumulative proportion
    - **RESPONSE: Revised to include the proportion by month in each era to the figure.**
40- Lines 363-364. This implies a major reduction in the impact of fishing on these populations, and therefore potential achievement of the conservation objectives. That is, unless there was also a 10-fold decline in the cod and yellowtail populations. Another additional way to state the degree of discard would be to report the proportion of the populations lost due to discarding in the scallop fishery. This would provide the missing information on the impact of discards to the populations.
    - **RESPONSE: See Response to Comment 22 about the conservation objectives of these closures.**
41- Lines 366-368 It appears it is above average for the three months after the closure for cod and three months before and after for yellowtail.
    - **RESPONSE : Agreed, here we wanted to keep it simple to show that discards are elevated in the months adjacent to the closure for both species and emphasize that the closure duration is too short on each side of both closures.**
42- Figure 8 Why do most of the observed data points, way more than 5%, fall outside of the 95% confidence interval? The data in c and e is the same, just transformed. Same for d and f. However, the curves fit in image c and e appear very different. Rather than attempting to fit a spline to these point that assumes some degree of correlation to neighboring months just show the medians for each month.
    - **RESPONSE: The data in c and e are actually somewhat different in how they are calculated, you can see in these figures that the highest values of the proportion of annual discards in a given month may not have the highest discard rate anomaly. The discard rate anomaly does not include information about the total discards, whereas the proportion of total discards does and this is what leads to the differences and is why these are included in these. Regarding the GAM se’s we can confirm these are the 95% CI’s estimated from the GAM model fits based on the link scale and then transformed to the natural scale for the figures, See** [**Marra and Wood 2012**](doi:%2010.1111/j.1467-9469.2011.00760.x) **for an interesting discussion of GAM CI’s.**
43- Lines 398-399 The results show fishing shifted to more productive areas during the closure era, a positive correlation between time-area closures and socio-economic objectives as expressed through the metric of effort allocated to more productive locations. The results show a 10-fold decrease in discard weight, and a seasonal pattern of discarding weight and rate. Neither provides information on the abundance or productivity of the populations of conservation concern. Therefore, because both the conservation objectives are not specified, and a reduction of discard weight from the scallop fishery presumably is the goal of closures in terms of achieving conservation, the results do not support the conclusion that time-area closures did not achieve conservation objectives.
    - **RESPONSE: See response to comment 22 in which the conservation objectives are clarified.**
44- Line 400-401 The methods here leave out a key step of specify objectives. Without clear objectives performance metrics that measure the status of those objectives will be lacking and monitoring may not measure the parameters of value.
    - **RESPONSE: See response to comment 22 in which the conservation objectives are clarified.**
45- Lines 420-423 Excellent point!
    - **RESPONSE: Thanks!**
46- Line 422 Add “in”, to obtain “…coincidental changes in the state…”
    - **RESPONSE: Good catch, thanks!**
47- Line 427-429: An important note!
    - **RESPONSE: Indeed!**
48- Line 433-435 This presumes the amount of discarding would have to be anomalous to be effective. This may not be the case because, 1) effective is not defined, and 2) the discarding that would have resulted without the closure is unknown. Perhaps there would have been an anomalously large amount of discarding during those time periods without the closures. Finally, in the case of yellowtail where fishing seemingly would occur outside of closures anyway, an objective of educating the fishing community about where and when yellowtail spawn to create a conservation mindset, or to practice should the population recover and therefore be subject to additional fishing pressure, could also be an objective and affect the conservation of the population in other ways.
    - **RESPONSE: By anomalous we are saying that there should be a signal in the discard data if the closures are achieving the conservation objectives as irrespective of other actions the discards observed in the scallop fishery during the month of the closure should decline. We agree that it is possible that during this month that perhaps there would have been very large discards and we state this in the middle of this same paragraph (Lines 438 -440 in original manuscript). Given that yellowtail and the 2 months of cod data indicate nothing unusual is happening in the months that the closures are in place then we believe this is relatively strong circumstantial evidence that discarding rates would not be anomalously large in these months if the closures were not in place. Also we note later in the discussion other actions by the fishery in which they are demonstrating a ‘conservation mindset’ which may well be more effective than these closures.**
49- Line 440-443: Enough and adequate are value statements that have not been defined and no information is provided about the spatial distribution of cod or yellowtail. This conclusion is not supported. The only conclusion that can be drawn from the data provided is that the closures do not eliminate by-catch and discarding of cod and yellowtail. However, that amount of reduction in by-catch may be more than enough or exceed what is adequate.
    - **RESPONSE: We have revised the wording, but we believe the results strongly support that the closures are not fully meeting their stated conservation objective of protecting spawning aggregations of either species. The rationale for this as outlined in the papers are:**
      i. **The closures do not reduce discards rates below levels observed in adjacent months when the species are still known to be spawning**
      ii. **The temporal extent of these closures is too short because discard rates are elevated in the months before and after the closure.**
      iii. **Given there is no fishing inside the closures, and that discard rates during the closure months are at or above the discard rates observed in the month immediately before and after the closures are in place, it is clear that these closures cannot be accounting for the full spatial extent of the spawning aggregations. What else would the fishery be catching during spawning other than these spawning aggregations which are outside the closure boundaries?**
50- Line 465-466: Declines in yellowtail biomass
    - **RESPONSE: Good catch, thanks!**
51- Lines 457 “Despite incomplete elimination of discarding during these closures, …
    - ”**RESPONSE: Revised**
52- Line 458-459 Yes.
    - **RESPONSE: We have also observed a similar decline in discards of other groundfish during this period from this fishery.**
53- Line 459-466 How low is the biomass of these populations, and have they declined by an order of magnitude? Discards below the quota seems like an indicator of closure success rather than an indicator of a population’s conservation status.
    - **RESPONSE: The Yellowtail decline during this period has likely been very large, but there is currently no quantitative stock assessment model for either of these stocks at this time. See evidence later for similar declines in yellowtail bycatch from the US scallop fishery during this same period.**
54- Line 492-494 Remove inaccurate concluding sentence, or restate that extending the duration of the closures may reduce by-catch by capturing a greater portion of the period when cod and yellowtail populations occupy Georges Bank.
    - **RESPONSE: See response to Comment 49.**
55- Line 495-496 “To be maximally effective…
    - **RESPONSE: Upon review, we prefer the original wording**
56- Line 496-497 Remove. Without a pre-post comparison the impact on the discard risk is unknown. Alternatively, have not … eliminated the discard risk.
    - **RESPONSE: Unclear what lines are being referred to here**
57- Line 497-499 Therefore, because these populations have not achieved conservation targets
    - **RESPONSE: Unclear what lines are being referred to here**
58- Line 521-525 This information is crucial to interpreting the discard data presented in the results and should not be withheld until the final sentence of the discussion. For example, if the effort from pre to post closure reduced by 50%, then the 10-fold reduction in discard weight is a 5-fold reduction in discard rate per unit of effort.
    - **RESPONSE: We have added this information earlier in the discussion when first discussing the order of magnitude decline in discards.**
59- Line 527-529. Remove “conservation impact”, or rephrase as “the degree of discarding unaffected by the current closure practices”
    - **RESPONSE: Revised to align with Comment 22 revisions**
60- Line 528-529 “establishment of a dedicated to monitoring program”
    - **RESPONSE: Revised**
61- Line 529-531 Remove “the spatial and temporal extent of these closures were insufficient to protect either species during spawning and” or rephrases as “insufficient to eliminate by-catch”
    - **RESPONSE: Revised**
62- Line 531 replace “significant” with “consequential” or the like
    - **RESPONSE: Revised**
63- Line 532-533 What is the evidence for this?
    - **RESPONSE: The evidence includes:**
      i. **The continued elevated discard rates during the closures**
      ii. **The majority of the discards during the year occurring in the months before, during, and after the closures**
      iii. **The significant reduction in effort from the scallop fishery**
      iv. **Similar declines in bycatch of Yellowtail in the US scallop fishery.**
