# Supplementary material for: Evaluating socio-economic and conservation impacts of management: A case study of time-area closures on Georges Bank
Source: PLoS One. 2020 Oct 13;15(10):e0240322. doi: 10.1371/journal.pone.0240322 (PMC7553282; doi:10.1371/journal.pone.0240322)
Supplement: Attachment — Submitted filename: Response to Reviewers.docx [file pone.0240322.s003.docx]

Response to Reviewers

Page 18, Lines 413-414 - Rephrase this sentence to clarify that the *conservation* objectives may not be met by the closures. Perhaps a simple change like this will do: "We found that time-area closures had minimal impact on the scallop fishery, *however*, results suggest that the *conservation* objectives of the closure are not being fully realized.

Thank you Revised

Page 19, Lines 435-437 - Consider rephrasing this sentence as well. Should "ineffective" be "effective"? I think this paragraph could start with something simple, like this: "A lack of direct monitoring data poses challenges to evaluating the effectiveness of time-area closures" because monitoring data is important for evaluating socio-economic and conservation objectives.

Thank you Revised

Reviewer #2: The authors have responded to my concerns and I can confidently respond affirmatively to the PLOS One review criteria (apart from the data availability question with is due to sharing restrictions). I thank the authors for the completeness of their responses, in particular to the nuanced methodical and results questions. Thank you for carefully addressing the semantic confusion around conservation objectives in the original manuscript submission. I hope that will avoid confusion for future readers and broaden the audience the manuscript reaches. If the authors are not familiar with value of information analyses (e.g. Runge et al 2011 Which uncertainty? Using expert elicitation and expected value of information to design an adaptive program) I encourage them to explore this methodology and consider it for future evaluations of this or other similar data for us in a management context.

Thank you
